# *CDC73* gene mutations in sporadic ossifying fibroma of the jaws

**DOI:** 10.1186/s13000-016-0532-0

**Published:** 2016-09-22

**Authors:** Yan Chen, Da-Yan Hu, Ting-Ting Wang, Ran Zhang, Qing Dong, Zhi-Xiu Xu, Lin Wang, Tie-Jun Li

**Affiliations:** 1 Department of Oral Pathology, Peking University School and Hospital of Stomatology, 22 South Zhongguancun Avenue, Haidian District, Beijing, 100081 People’s Republic of China; 2 Department of Oral Medicine, North China University of Science and Technology, School and Hospital of Stomatology, 82 South Construction Road, Lubei District, Tangshan, 063000 People’s Republic of China; 3 Department of Stomatology, The First Affiliated Hospital of Bengbu Medical College, 287 Changhuai Road, Bengbu, 233004 People’s Republic of China

**Keywords:** *CDC73*, Ossifying fibroma, Sporadic

## Abstract

**Background:**

The tumor suppressor gene *CDC73* was found to be associated with hyperparathyroidism-jaw tumor syndrome (HPT-JT), which is characterized by parathyroid adenoma or carcinoma, ossifying fibroma (OF) of the jaws, and renal and uterine lesions. Mutations in *CDC73* have also been frequently detected in sporadic parathyroid carcinomas and renal tumors. However, the prevalence and range of *CDC73* mutations in sporadic OFs have not been established.

**Methods:**

We directly sequenced coding and flanking splice junctional regions of *CDC73* in 40 cases of sporadic OF of the jaws. We also used immunohistochemistry to detect parafibromin, the protein product of *CDC73*, in those cases.

**Results:**

Two novel *CDC73* mutations were identified in 2 of the 40 cases (5 %). Both were somatic mutations located in exon 1 of the coding region. Strong parafibromin expression was detected in all 40 cases, irrespective of the presence of *CDC73* mutations.

**Conclusions:**

Mutations in*CDC73* were rare in sporadic OF of the jaws, but may affect the pathogenesis of a small subset of tumors of this type.

## Background

According to the World Health Organization, it is proposed that benign fibro-osseous lesions be divided into 3 categories, including fibrous dysplasia, ossifying fibroma, and osseous dysplasia [1, 2]. The most common forms of BFOL are ossifying fibroma (OF) and fibrous dysplasia (FD) and they manifest different clinical courses and should be treated differently [3].

Ossifying fibroma is a benign tumor thought to arise from the periodontal ligament, which potential of continuous growth if not treated [4, 5]. It is a slow-growing, generally lack of symptoms, but can cause serious cosmetic and functional problems. [6] OF can occur in almost any bone in the craniofacial region, predominantly in the premolar-molar region of the mandible, with adult women more frequently affected [7]. Radio-graphically, OF usually presents a well-circumscribed radiolucency with varying degrees of calcification that rarely erodes or displaces teeth [4]. Ossifying fibromas are typically encountered as solitary lesions, multiple and/or familial lesions rarely present in clinic [8]. In some instances, OF can be destructive and risk for recurrence, as a result of which completely surgical enucleating is needed [9].

FDs are genetic and non-inheritable, caused by missense mutations that occur post-zygotically in the gene, *GNAS,* which is located on chromosome 20q13 and codes for the a-subunit of the stimulatory G-protein, Gs [10]. The mutations generate a somatic mosaic and the resulting proteins display reduced GTPase activity and, consequently, increased activation of adenylyl cyclase. Therefore, mutated cells constitutively generate high levels of cAMP and have a high rate of proliferation [11]. The differential diagnostic value of *GNAS* in FD is well recognized. In previous studies, our research group demonstrated that, in a total of 307 cases of FD and 23 cases of OF, the mutation rate of *GNAS* in patients with FD was approximately 86 % (264/307), while *GNAS* mutations were not found in patients with OF. [12] Little is known about the etiology of OF.

Hyperparathyroidism-jaw tumor (HPT-JT) syndrome is an autosomal dominant, multiple neoplasia syndrome which is characterized by parathyroid adenoma or carcinoma, ossifying fibroma of the jaws, renal and uterine lesions [13, 14]. Between 30 and 40 % of individuals with HPT-JT also develop OFs, which are distinct from the “brown” tumors associated with severe hyperparathyroidism.. The HPT-JT locus was mapped to chromosome 1q24–q32; the putative gene, designated first as hyperparathyroidism type 2 (*HRPT2)* and then as *CDC73*, encodes a 531 amino acid protein called parafibromin. [15] Parafibromin is ubiquitously expressed and is evolutionarily conserved. It is the human homologue of yeast Cdc73, which is a component of the yeast RNA polymerase II/Paf1 complex that is important for histone modification and post-transcriptional events [16, 17]. Mutations in *CDC73* can be detected in approximately 58 % of probands with clinical features of HPT-JT syndrome [15]. In one series, which included four cases with OFs, mutations in *CDC73* were found in two of the cases [18].

Collectively, these data suggested that *CDC73* may be a key factor in the etiology of HPT–JT syndrome and its related tumors, including OFs. The prevalence and range of *CDC73* mutations in sporadic OFs remain to be determined, in view of the limited number of cases examined to date. The goal of the present study was to identify *CDC73* mutations in a group of Chinese patients presenting with sporadic OFs and to examine the possible role of *CDC73* in the pathogenesis and diagnosis of OFs.

## Methods

### Subjects and samples

The fresh tumor specimens and peripheral blood samples of 40 sporadic OFs were obtained from the Department of Oral Pathology, Peking University Hospital and School of Stomatology, during 2003–2015. The diagnosis was made according to the WHO classification of odontogenic tumors [2]. Samples were only collected if the patient’s family history was negative and PTH serum level was normal, to exclude cases of HPT-JT related OF. Detailed information regarding these cases is provided in Table 1.

**Table 1:** Clinical data and *CDC73* mutation of 40 patients with sporadic ossifying fibromas

Patient	Age (y)	Gender	Location	Symptoms and signs	Radiographic findings		*CDC73* mutation
1	10	female	right maxilla	right facial swelling bite pain	Mixed lesion	well demarcated	c.13-16delCTTA
2	18	female	left mandible	left posterior teeth discomfort	Mixed lesion	well demarcated	c.8-10delACGinsCT
3	23	female	left mandible	bone swelling	Mixed lesion	ill demarcated	No mutation
4	18	female	right mandible	right facial swelling occasional discomfort	Mixed lesion	well demarcated	No mutation
5	20	female	left maxilla	left facial swelling surface fester	Mixed lesion	well demarcated	No mutation
6	46	female	left mandible	bone swelling numbness of lower lip	unavailable	unavailable	No mutation
7	39	female	left mandible	bone swelling	Radiolucent	well demarcated	No mutation
8	1	male	right mandible	right facial swelling	unavailable	unavailable	No mutation
9	14	male	left mandible	bone swelling	Radio-opaque	well demarcated	No mutation
10	34	male	left mandible	left facial swelling	Radio-opaque	well demarcated	No mutation
11	10	male	left maxilla	right facial swelling tinnitus	Radiolucent	well demarcated	No mutation
12	43	female	anterior maxillary region	bone swelling	Radio-opaque	well demarcated	No mutation
13	15	male	left mandible	bone swelling	Mixed lesion	well demarcated	No mutation
14	22	female	left maxilla	bone swelling	Radio-opaque	well demarcated	No mutation
15	31	female	right mandible	buccal and lingual bone expansion mildly pain	Mixed lesion	well demarcated	No mutation
16	32	female	left mandible	buccal and lingual bone expansion	Mixed lesion	well demarcated	No mutation
17	37	male	right maxilla	right facial swelling pain and fever	Mixed lesion	well demarcated	No mutation
18	43	female	right maxilla	buccal bone expansion	Mixed lesion	well demarcated	No mutation
19	47	male	right maxilla	bone swelling nasal obstruction	Mixed lesion	ill demarcated	No mutation
20	6	male	right maxilla	bone swelling	Mixed lesion	well demarcated	No mutation
21	9	male	right mandible	right facial swelling	Radiolucent	well demarcated	No mutation
22	24	female	anterior mandibular region	pain	Mixed lesion	well demarcated	No mutation
23	26	male	left maxilla	buccal bone expansion	Mixed lesion	well demarcated	No mutation
24	10	male	left maxilla	left facial swelling	Mixed lesion	well demarcated	No mutation
25	11	male	right maxilla	nasal obstruction	Mixed lesion	well demarcated	No mutation
26	5	male	left maxilla	bone swelling	Mixed lesion	well demarcated	No mutation
27	44	female	right maxilla	sense of numbness	Mixed lesion	ill demarcated	No mutation
28	31	female	left mandible	bone swelling looseness of teeth	Radiolucent	well demarcated	No mutation
29	17	male	right maxilla	bone swelling	Mixed lesion	ill demarcated	No mutation
30	20	female	right mandible	bone swelling occasional pain	Mixed lesion	well demarcated	No mutation
31	6	male	left mandible	bone swelling	Radiolucent	ill demarcated	No mutation
32	11	female	right mandible	right facial swelling	Mixed lesion	well demarcated	No mutation
33	14	male	anterior mandibular region	labial bone expansion	Radiolucent	ill demarcated	No mutation
34	20	female	left maxilla	bone swelling	Radio-opaque	well demarcated	No mutation
35	21	female	right mandible	bone swelling	Radiolucent	ill demarcated	No mutation
36	7	male	right mandible	bone swelling numbness of lower lip	Mixed lesion	ill demarcated	No mutation
37	8	male	left maxilla	left facial swelling	Mixed lesion	ill demarcated	No mutation
38	27	female	left mandible	bone swelling	Mixed lesion	well demarcated	No mutation
39	19	female	Les.1:right maxilla	hard bone swelling	Radiolucent	well demarcated	No mutation
			Les.2:bilateral mandible				
40	6	male	Les.1: right maxilla	bone swelling	Mixed lesion	well demarcated	No mutation
			Les.2: left maxilla				
			Les.3: right mandible				
			Les.4: left mandible				

### DNA Extraction and Polymerase Chain Reaction (PCR)

Genomic DNA was extracted from frozen samples (25 mg) of neoplastic tissue and peripheral blood by using a DNeasy Tissue Kit (Qiagen Sciences, Maryland, USA). The 17 coding exons of *CDC73* were amplified as 15 different fragments with primers derived from the flanking intronic or 3′/5′UTR regions, to allow the detection of mutations that occurred in coding regions or that affected splicing, as previously described [19]. The condition of PCR was used as follows: initial denaturation at 95°C for 5 min; 35 cycles of denaturation at 95 °C for 30 s, annealing at 58–62 °C for 30 s, elongation at 72 °C for 30 s; and a final extension at 72 °C for 7 min.

### Direct sequencing

The amplified PCR products were gel-purified and directly sequenced. When nucleotides insertion or deletion was detected, clone sequencing by the plasmid vector was used for confirmation. The method of clone sequencing were carried out by previously described [20]. All detected mutations were confirmed by reverse sequencing and at least two independent experiments.

### Immunohistochemistry

Parafibromin expression was evaluated in formalin-fixed and paraffin-embedded tissues by immunohistochemical staining as previously described [21], using a mouse monoclonal anti-parafibromin antibody (SC-33638, Santa Cruz Biotechnology Inc., Santa, Cruz, CA, USA) that recognizes amino acids 87–100.

## Results

### Clinicopathological features

The clinical characteristics of the 40 cases enrolled in this study are summarized in Table 1. The patient age at first presentation ranged from 1 to 47 years (median age: 19.5 years). The male to female ratio was 19:21. Seventeen (44.7 %, 17/38) tumors occurred in the maxilla, while 21 (55.3 %, 21/38) occurred in the mandible. Two cases, which have been reported previously [8], showed multiple lesions affecting both the maxilla and the mandible. Bone or facial swelling (90 %, 36/40) was the most common clinical presentation, and other presentations included pain (10 %, 4/40), bite pain (2.5 %, 1/40), nasal obstruction (5 %, 2/40), sense of numbness (7.5 %, 3/40), sense of discomfort (5 %, 2/40), looseness of teeth (2.5 %, 1/40), tinnitus (2.5 %, 1/40), and surface fester (2.5 %, 1/40). In 38 of 40 patients with available radiographs, radiographic features included radiolucent (eight cases, 21 %), mixed (25 cases, 66 %;), and radioopaque lesions (5 cases, 13 %). Lesions of 29 cases were well-defined, and those of 9 cases were ill-defined.

### *CDC73* mutations

**Fig. 1:**
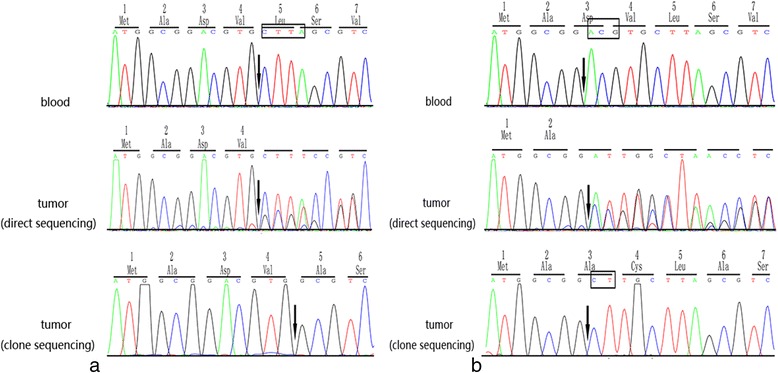
Two novel somatic mutations of *CDC73* identified in sporadic ossifying fibromas. Sequences of both peripheral blood (upper) showing the wild-type sequence and tumor (medium: directly sequencing. Lower: clone sequencing) are shown. **a** In case 1, c.13_16delCTTA (highlighted in small box), which leads to a frame shifting change with the fifth amino acid residue leucine as the first affected amino acid, changing into a alanine. **b** The other novel mutation, c.8_10delACGinsCT (highlighted in small box), was found in case 2. The frame shifted from the third amino acid residue aspartic acid that is changed into alanine

Results from the mutational analysis of *CDC73* in 40 cases of OF are summarized in Table 1. Two novel *CDC73* mutations were identified in two cases. Both mutations were somatic and located in exon 1. The mutation in case 1 was a frameshift mutation (c.13_16delCTTA), which leads to a premature stop codon at position 15. The frameshift mutation also causes the substitution of the fifth amino acid, leucine, which was the first affected amino acid, with an alanine (p.Leu5Alafs*15). The novel mutation found in case 2, c.8_10delACGinsCT, was a frameshift mutation that leads to a premature stop codon at position 18. This mutation causes the substitution of the third amino acid, aspartic acid, with an alanine (p.Asp3Alafs*18) (Fig. 1).

### Immunohistochemical detection of parafibromin expression

**Fig. 2:**
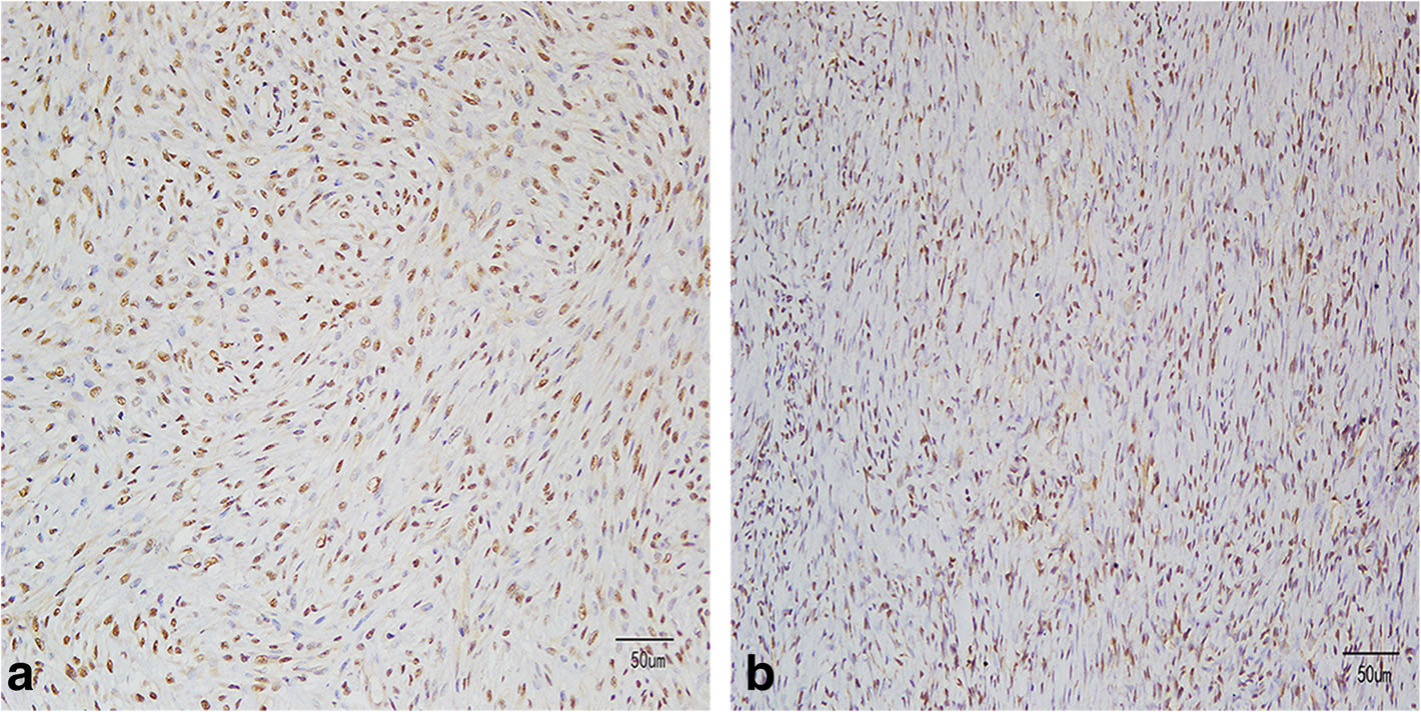
Parafibromin staining by immunohistochemistry. All tumors, without (**a**) or with (**b**) *CDC73 *mutation (*200), showed strong nuclear and cytoplasmic staining in both fibroblasts and osteoblasts within the lesion

Using immunohistochemical analysis, we found that all cases of sporadic OF showed strong parafibromin expression in the nucleus, and that some showed parafibromin expression in the cytoplasm of the spindle-shaped lesion cells, including those cells that were carriers of a *CDC73* mutation (Fig. 2).

## Discussion

Parafibromin, the product of *CDC73*, is believed to be important for embryonic development and tumorigenesis [15]. Homozygous *CDC73* knockout mutations in mice led to in utero death by stage E6.5. Conditional knockout of both *CDC73* alleles in E8.5 or older mice resulted in the retardation of embryonic growth and increased apoptosis, whereas in adult mice it resulted in cachexia and death within 20 days [22]. Parafibromin is a ubiquitously expressed nuclear protein [21]. In humans, parafibromin interacts with RNA polymerase II via the human PAF1 complex, which also includes human Paf1, CTR9, and Leo1. This complex regulates a number of key transcriptional events, including transcription initiation, transcript elongation, and post-transcriptional events, including mRNA maturation and maintenance of poly (A) tail length [16, 17].

Mutations in *CDC73* have been frequently detected in patients with HPT-JT and also occur in 20–29 % of individuals with apparently sporadic parathyroid carcinoma [23]. However, the frequency of *CDC73* mutations in sporadic parathyroid adenomas is low, at 0–4 %, indicating that *CDC73* mutations likely confer an aggressive growth potential and may result in malignant transformation of parathyroid cells [24]. *CDC73* mutations have been detected in between 0 and 33 % of individuals with familial isolated primary hyperparathyroidism [23]. Somatic *CDC73* mutations have also been found in sporadic renal tumors [25].

The frequency of *CDC73* mutations in individuals with sporadic OF of the jaws has not been extensively studied. In the present study, two somatic mutations were identified in 40 cases of OF (5 %). This result suggested a possible pathogenetic role for *CDC73* in some cases of sporadic OFs. Previously, a few cases of sporadic OF was found to harbor a mutation in *CDC73* [18, 26]. Further studies are needed to clarified the exact frequency of CDC73 mutation in sporadic OF due to limitation of sample size.

The *CDC73* mutations identified in this study included two novel somatic mutations (c.13_16delCTTA and c.8_10delACGinsCT), which caused frameshifts and premature truncations of the protein. This finding is consistent with previous studies showing that over 75 % of the reported *CDC73* mutations are frameshift or nonsense mutations that are predicted to result in either the truncation of the parafibromin protein or in loss of the translated protein through nonsense-mediated mRNA decay.

Somatic mutations of *CDC73* were predominantly located in exons 1 and 2 (55 and 21 %, respectively), in contrast to germline mutations (22 and 16 %, respectively) mainly detected in HPT-JT, which were predominantly located in exon 7 (30 %). Only one somatic mutation has been detected in exon 7 to date [24]. The two mutations that we identified in OF both occurred in exon 1, and a previous study on OF [18] also identified a somatic mutation in exon 1. The basis of the differences in the distribution of the germline and somatic *CDC73* mutations remains to be elucidated.

In vitro studies have shown that parafibromin acts as a tumor suppressor. Overexpression of parafibromin inhibits the proliferation of NIH3T3 and HEK293 cells, increases G1 arrest and apoptosis in HeLa cells, and downregulates expression of the cell cycle regulator, cyclin D1 [27–29]. Likewise, RNAi-mediated inhibition of parafibromin expression in HeLa cells resulted in increased S-phase entry with reduction in basal apoptosis and increase in expression of the proto-oncogene, c-myc [30, 31].

According to Knudson’s two-hit model for tumor suppressor genes [32] two mutations, one occurring in each of the two alleles of a gene, or one mutation in one allele of a tumor suppressor gene accompanied by the allelic loss of the remaining wild-type allele, are required to trigger neoplasm formation. Biallelic inactivation in *CDC73* has been detected in tumors in HPT-JT kindred and in sporadic parathyroid carcinomas and renal carcinomas [15]. Since parafibromin is ubiquitously expressed, biallelic inactivation of *CDC73* can be detected immunohistochemically by the diffuse loss of nuclear expression. This is a common feature in parathyroid carcinoma [33, 34].

However, the two-hit model may not apply to sporadic OF. First, only one mutation was detected in each case, even in the case with two somatic mutations reported by Pimenta [18]. Second, normal parafibromin expression was detected by immunohistochemistry. The effect of mutated *CDC73* on sporadic OFs needs to be studied further.

## Conclusions

Although *CDC73* mutations are rare in sporadic OFs of the jaws, *CDC73* may play a role in the pathogenesis of a small subset of tumors.

## Abbreviations

BFOL: Benign fibro-osseous lesion
FD: Fibrous dysplasia
HPT-JT: Hyperparathyroidism-jaw tumor syndrome
OF: Ossifying fibroma
PTH: Parathyroid hormone
